# The Best Medication

**Published:** 1859-10

**Authors:** 


### THE BEST MEDICATION.

Educated physicians of all schools unite in the opinion that a very large proportion of all ordinary ailments arise from injudicious eating—from eating too much. It seems to be a law of the vegetable kingdom, that if injury is done to a plant, or bush, or tree, it will recover itself, if let alone: if a mere animal is injured, it will recover itself if let alone, because both the vegetable and the animal are created with instincts which are the means of their preservation. Man is made under the same general law; he has this same instinct and being "more excellent than they"—has also vouchsafed to him the higher guard of reason, to enable him the more certainly to live until the grand aim of his creation has been attained, to wit, a fit preparation for an immortal existence. But our nature has been perverted, and, in the matter of preserving health and life, reason is dethroned, instinct is silenced, and mere animal gratification rules with desolating sway.

We all admit that we eat too much. The reader will most probably confess with great frankness, "I know I eat too much." And just as well does the physician know that this over-eating is a great cause of human sickness and suffering. When a man is ailing, he should, like the animal and vegetable, be let alone; which means, first, that the cause of his ailment should cease to operate; and, second, that he should let himself alone—that is, avoid eating, and do nothing. Let him lie down and rest as the brute will do, and nature will come to his aid with wonderful promptness and efficiency.

The best panacea on earth—that which has the best claim to the title of Catholicon, literally, "Cure All"—is abstinence from food. But, to be efficient, this remedy must be promptly applied—applied on the instant the knowledge of mischief presents itself. The great general rule is:

The very moment a man becomes conscious of any uncomfortable sensation about the body, let him take not an atom of food or anything else until that sensation has entirely disappeared. This simple prescription, promptly and resolutely followed, would save multitudes of lives every year, and would prevent an amount of human suffering which many figures would fail to express.

To make this rule more widely applicable, a few modifications may be added.

Although food is abstained from so advantageously, water may be drank most freely in the great majority of cases.

In most instances, repose, bodily quietude, should be specially observed during abstinence from food, for to exercise under actual hunger is unnatural; besides, animals, when they are ailing, are still: then nature employs all her powers in repair, instead of dividing them in locomotion.

If, by the next morning, there is but a partial abatement, some warm drink, with toasted bread or other light food, should be taken, but nothing more until noon, when a physician should be sent for, unless there is a decided abatement of ailings.

If, under abstinence, there is no abatement of symptoms within ten hours, medical advice should be taken.

It is not generally safe for any one to practice entire abstinence over forty-eight hours on his own responsibility, nor to practice a very low or a very scant diet for more than four or five days, unless by the advice of a physician; for sometimes persons have, by too great abstinence, brought on themselves a debility scarcely, if ever, to be recovered from. Abstinence is a most valuable remedy, but, if carried beyond reasonable limits, it is an agent of great mischief.

